# Neuropsychiatric Symptoms and Quality of Life in Patients With Adult-Onset Idiopathic Focal Dystonia and Essential Tremor

**DOI:** 10.3389/fneur.2020.01030

**Published:** 2020-09-11

**Authors:** Sangjin Lee, Sun Ju Chung, Hae-Won Shin

**Affiliations:** ^1^Department of Neurology, Chung-Ang University College of Medicine, Seoul, South Korea; ^2^Department of Neurology, Asan Medical Center, Ulsan University College of Medicine, Seoul, South Korea

**Keywords:** focal dystonia, essential tremor, neuropsychiatry, non-motor manifestations, quality of life

## Abstract

**Background:** While idiopathic focal dystonia (IFD) and essential tremor (ET) have been considered pure movement disorders, they reportedly induce neuropsychiatric manifestations and may thus be more accurately described as network disorders.

**Methods:** The present multi-center, cross-sectional, case-control study evaluated the severity of depression and anxiety with the Beck Depression Inventory (BDI) and Beck Anxiety Inventory (BAI), respectively; the frequency of neuropsychiatric disorders with the Korean-Mini International Neuropsychiatry Interview; and QoL with the Short-Form 36 (SF-36).

**Results:** Seventy-four subjects participated in this study (IFD, 27; ET, 24; controls, 23). The BDI and BAI scores were higher in the IFD and ET groups than in the control group. Although the frequency of neuropsychiatric disorders diagnosed according to the Diagnostic and Statistical Manual of Mental Disorders, 4th Edition Axis I was comparable among the groups, the prevalence of major depressive disorder tended to be high among patients with IFD. QoL was correlated with the severity of depression and anxiety across the groups.

**Conclusions:** Depression and anxiety are more severe in patients with IFD and ET compared to healthy controls, while their severity is similar among patients with IFD and ET. Axis I major depressive disorder is relatively more prevalent in patients with IFD. Neuropsychiatric symptoms affect QoL regardless of the affected individual's condition, addressing neuropsychiatric symptoms in patients with movement disorders may be crucial to improving their QoL.

## Introduction

Dystonia has traditionally been classified as a movement disorder, caused by dysfunction in the motor system, particularly the basal ganglia (BG) (1, 2). As the BG are connected to the limbic system, its dysfunction induces consequent non-motor neuropsychiatric symptoms in patients with dystonia (3–5). Indeed, recent studies have shown that such neuropsychiatric manifestations, including depression, anxiety, and obsessive-compulsive traits, are observed in patients with focal or segmental dystonia (6–15). The recent finding that patients with essential tremor (ET), a common motor characterized dysfunction of the cerebellum and consequent isolated tremor, also present with neuropsychiatric symptoms, may implicate psychiatric manifestations as part of the primary pathophysiology of movement disorders (3, 16–20).

However, no study has considered this possibility or compared the prevalence of psychiatric symptoms between patients with different movement disorders. The increasing body of evidence has shown a close correlation between the QoL of patients with movement disorders and their presentation of neuropsychiatric symptoms (21, 22). Furthermore, evaluating differences in the presentation and prevalence of non-motor neuropsychiatric symptoms across movement disorders would help gain an insight into their pathophysiology and improve the patients' QoL. The present study thus evaluated the frequency and severity of neuropsychiatric manifestations in patients with idiopathic focal dystonia (IFD) and ET, relative to healthy controls, and whether the considered neuropsychiatric symptoms affect the QoL in these subjects.

## Materials and Methods

This prospective multi-center, cross-sectional, case-control study was approved by the Institutional Review Boards of Chung-Ang University Hospital (C2013231-1191) and Asan Medical Center (2014-0149). All participants provided written informed consent before participation.

### Participants

The present study recruited patients with IFD, patients with ET, and age-matched, healthy controls without any neurological deficits. The sample sizes obtained using a power analysis were 43, 43, and 22 for patients with IFD, patients with ET, and controls, respectively. Diagnoses of IFD and ET were confirmed according to the Movement Disorder Society diagnostic criteria (23, 24). Specifically, among patients with ET, only those with tremor in both arms and without head tremor were enrolled to avoid the inclusion of patients with dystonic tremor. We excluded patients who presented with movement disorders other than dystonia or tremor or had a history of drug exposure to which the movement disorder could possibly be ascribed. None of the study subjects had been diagnosed with any psychiatric disorder, and all participants provided written informed consent prior to their enrollment in the study.

### Clinical Measurements

All participants were interviewed to evaluate the severity of their depression and anxiety using the Beck Depression Inventory (BDI) and Beck Anxiety Inventory (BAI), respectively (25, 26). They were also asked to undergo the Korean-Mini International Neuropsychiatry Interview (MINI). MINI is a brief, reliable structured interview used for diagnosing Axis I psychiatric disorders according to the Diagnostic and Statistical Manual of Mental Disorders, 4th Edition (DSM-IV) (27). The cognitive function of all participants was tested with the Korean-Mini Mental Status Examination (K-MMSE). The health-related QoL of participants was evaluated with the 36-Item Short-Form Health Survey (SF-36) (28). The Burke-Fan-Marsden Dystonia Rating Scale (BFMDRS) was used to evaluate the severity of dystonia in patients with IFD (29).

### Statistical Analysis

The Kolmogorov-Smirnov and Shapiro-Wilk tests were used to test the normality of the variables; we found that the variables analyzed by analysis of variance did not follow a normal distribution. Accordingly, The Kruskal-Wallis H and Mann-Whitney *post-hoc* tests were used to compare parameters, including BDI, BAI, SF-36, K-MMSE, and age, among the three groups. The chi-square test was used to compare the frequency of psychiatric disorders between groups. Linear regression was performed to determine the correlation between the severity of non-motor scores, including BDI and BAI, and scores of SF-36. The correlation between BFMDRS and scores of SF-36 was also analyzed using linear regression. Data are expressed as mean ± SD, and *p* < 0.05 were considered to indicate statistical significance. The data were analyzed using SPSS 25.0 statistical software (IBM Corp., Armonk, NY, USA).

## Results

We recruited 27 patients with IFD, 24 patients with ET, and 23 healthy controls. Of the patients with IFD, 13 had cervical dystonia; 2, focal hand dystonia; and 11, cranial dystonia. The demographic and clinical characteristics of the subjects are described in Table 1. There were no significant differences in terms of age, sex, or K-MMSE score between the groups (Table 1). The mean BFMDRS in the IFD group was 7.04 ± 6.51 (mean movement score, 6.11 ± 5.33; mean disability score, 0.93 ± 1.63). Symptom duration was comparable between patients with IFD and ET (100.21 ± 140.64 and 119.25 ± 91.14 months, respectively).

**Table 1:** Demographic and clinical characteristics of participants.

**Group (number of subjects)**	**Idiopathic focal dystonia (27)**	**Essential tremor (24)**	**Controls (23)**	***p*-value**
Age (year)	58.79 ± 15.18	65.58 ± 10.96	55.82 ± 14.93	0.5712
Symptom duration (months)	100.21 ± 140.64	119.25 ± 91.14	–	0.5720
K-MMSE	27.21 ± 2.19	25.67 ± 4.74	26.93 ± 2.31	0.8508
BFMDRS	4.04 ± 3.71	–	–	–
- Movement score	3.75 ± 3.43	–	–	–
- Disability score	0.29 ± 0.71	–	–	–

*Data are presented as mean ± SD unless stated otherwise. BFMDRS, Burke-Fahn-Marsden Dystonia Rating Scale; K-MMSE, Korean-Mini Mental Status Examination*.

**Figure 1:**
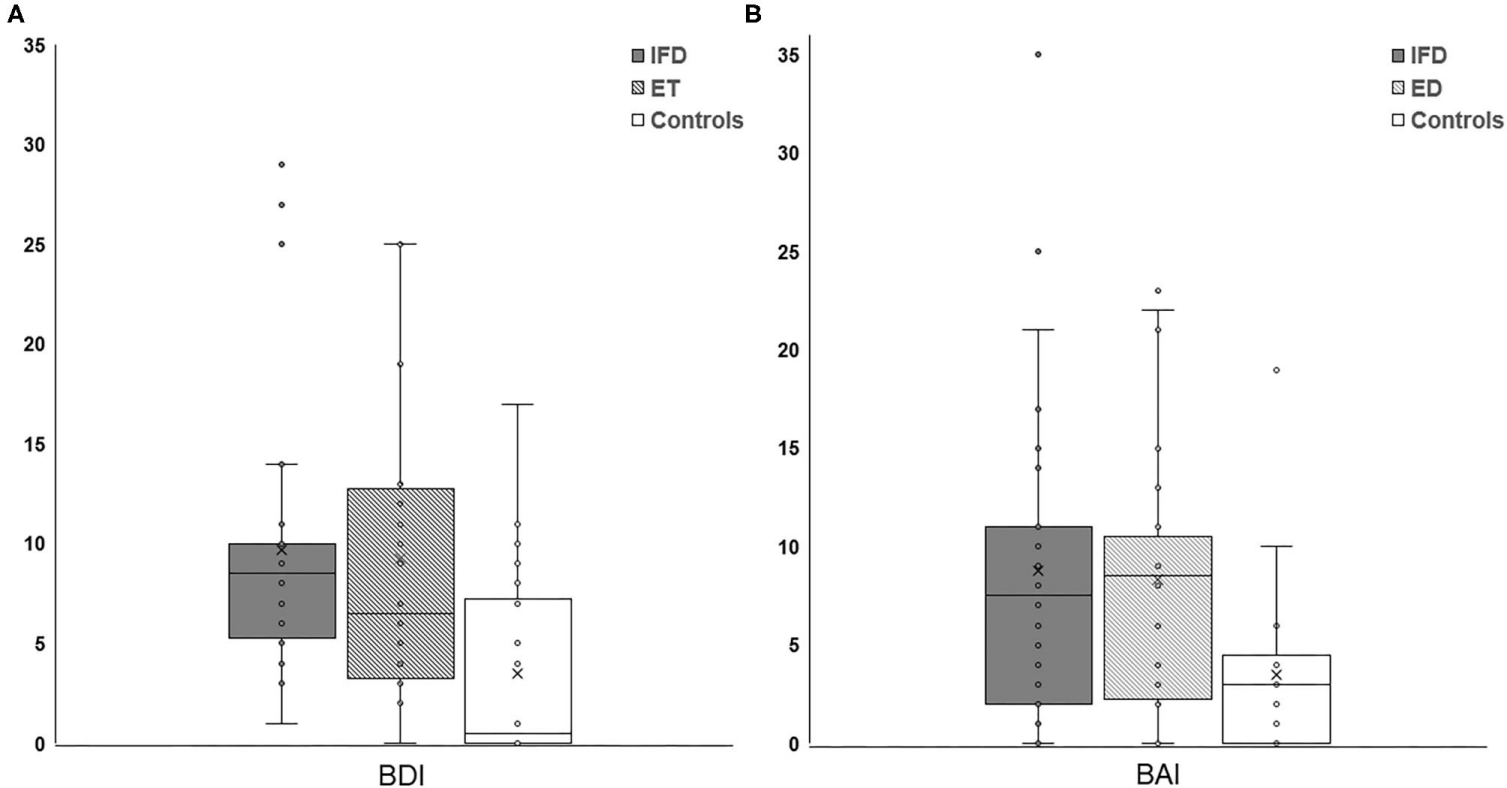
Comparison of the severity of depression **(A)** and anxiety **(B)** between individuals with IFD and ET and controls. The depression and anxiety scores were higher among patients with IFD and ET than in controls but comparable between patients with IFD and ET. IFD, idiopathic focal dystonia; ET, essential tremor.

The Kruskal-Wallis *H*-test showed that there was a significant difference in BDI and BAI among the groups (Kruskal-Wallis *H*-value = 13.018, 8.261; *df* = 2, 2; *p* = 0.001, 0.016, respectively). The Mann-Whitney *post-hoc* comparison revealed that patients with IFD and ET had higher BDI and BAI scores than healthy controls (IFD, ET, and control groups: 9.71 ± 6.85, 9.25 ± 7.40, and 3.85 ± 4.96, respectively, for the BDI score; Figure 1A, *p* = 0.001 and *p* = 0.013; 8.79 ± 8.12, 8.33 ± 6.60, and 3.80 ± 4.40, respectively, for the BAI score; Figure 1B, *p* = 0.004 and *p* = 0.010). We found no significant difference in the BDI or BAI scores between the IFD and ET groups (*p* = 0.525 and 0.993, respectively; Figure 1). Additionally, no significant differences were found in either the BDI or BAI scores among IFD types.

**Figure 2:**
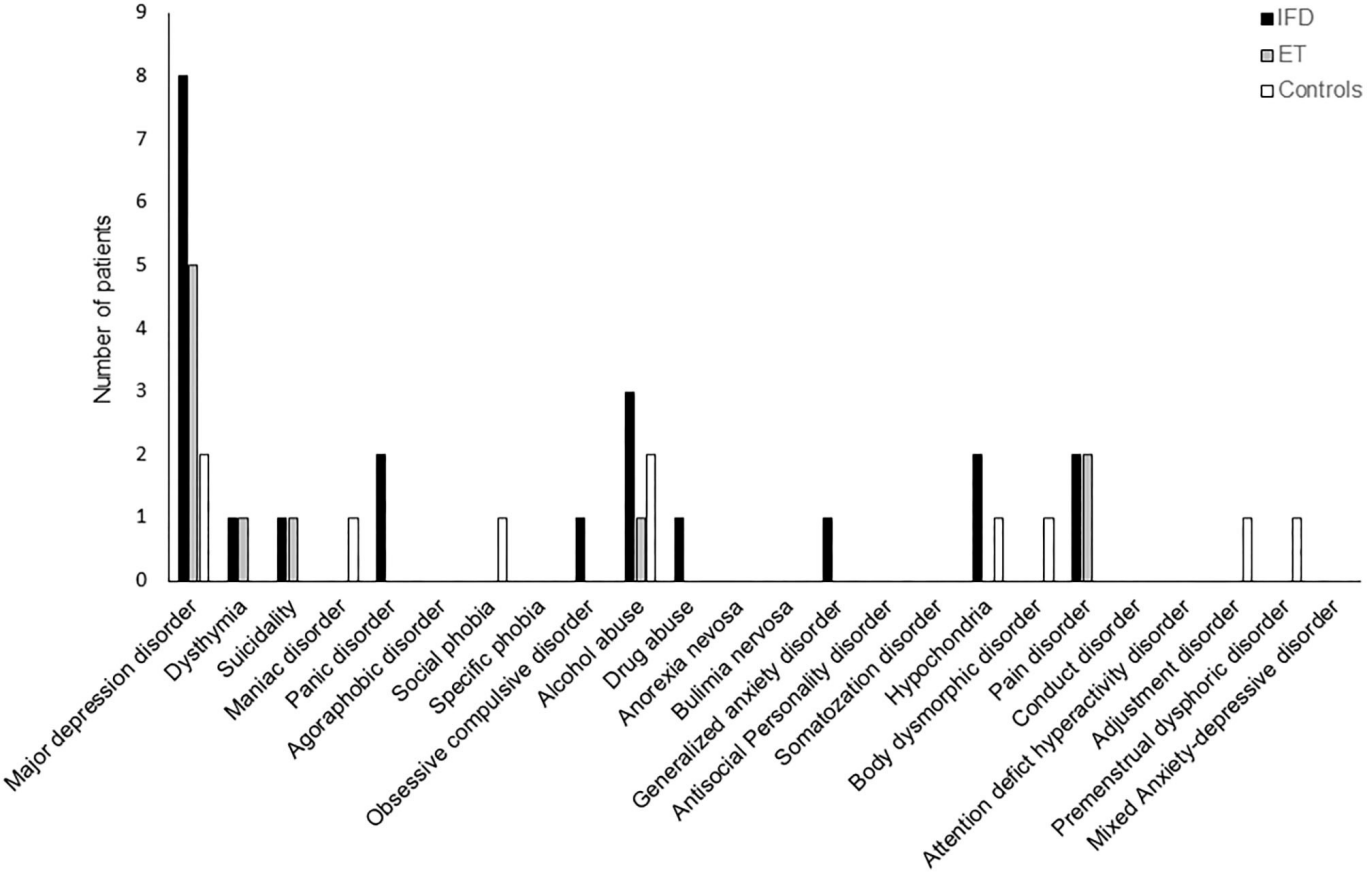
The frequency of DSM-IV Axis I neuropsychiatric disorders among individuals with IFD and ET and controls. The overall frequency was comparable among the groups, but the incidence of major depressive disorder tended to be higher in patients with IFD.

The frequency of Axis-I psychiatric disorders was comparable across the groups (*p* = 0.111); however, patients with IFD tended to have a higher frequency of occurrence of psychiatric disorders (48, 33, and 17% in the dystonia, ET, and control groups, respectively; Figure 2).

There was significant difference in SF-36 score among groups (Kruskal-Wallis *H*-value = 13.918, *df* = 2, *p* = 0.001) Patients with IFD and ET showed lower SF-36 scores than those of healthy controls (110.21 ± 14.33, 109.04 ± 24.29, and 126.16 ± 12.89, respectively, *p* < 0.05). However, there was no significant difference in SF-36 scores between patients with IFD and ET (*p* = 0.734). The linear regression analysis showed that BDI and BAI scores were significantly correlated with SF-36 scores. Among patients with IFD, SF-36 scores were significantly correlated with both BDI and BAI scores but not with BFMDRS scores.

## Discussion

The principal findings of this study are as follows: (1) patients with IFD or ET presented higher levels of depression and anxiety than did healthy controls; (2) the severity of depression and anxiety did not differ significantly between patients with IFD and ET; (3) while psychiatric disorders were more common among patients with IFD, their prevalence did not significantly differ between patients with IFD and ET and healthy controls; (4) the severity of depression and anxiety was significantly correlated with QoL across all subjects; and (5) QoL was not correlated with motor symptoms in patients with IFD.

Our results replicate previous findings of a relatively higher prevalence and severity of anxiety and depression among patients with idiopathic dystonia or ET than in healthy controls (6, 8, 9, 11, 13–16, 18–20, 30). Although our study did not find a significant difference between the study groups in terms of the frequency of psychiatric disorders diagnosed according to the DSM-IV Axis I (*p* = 0.11), psychiatric disorders tended to be more prevalent among patients with IFD than among healthy controls. Along with higher depression and anxiety scores, the frequency of major depressive disorder tended to be higher in patients with IFD (8, 2, and 2 in IFD, ET, and healthy controls, respectively); however, on account of our limited sample size, statistical significance could not be confirmed.

The frequency with which Parkinson's disease (PD) induces depression and anxiety can be ascribed to the spread of neurodegeneration beyond the BG (31, 32), which is the key neural substrate of PD. Although dystonia is a movement disorder related to the BG, neuropsychiatric manifestations in dystonia have been overlooked because of the lack of a specific structural pathology in this condition (33, 34). Since sensory dysfunction was introduced in this motor system disorder (35, 36), other neuropsychiatric manifestations have been highlighted as features of dystonia, particularly in the past decade (10–15). Furthermore, emerging evidence suggests that dystonia results from dysfunctional brain networks (37, 38). According to this hypothesis, the neuropsychiatric manifestations of dystonia can be considered primary consequences of the disrupted connection between the limbic and BG-motor symptoms in dystonia (7, 33, 39). ET could cause mood disorders and psychiatric symptoms by this same mechanism (4). Indeed, cross-sectional and longitudinal studies have reported an association between ET and non-motor symptoms (18). When ET is considered a neurodegenerative disorder such as PD, neuropsychiatric manifestations can be regarded as a consequence of pathological spread beyond the cerebellum, resulting in various symptoms besides tremor (40). On the other hand, the cerebellum is linked to the BG, and this thus provides a mechanism by which dysfunction of the cerebellum could affect the BG circuit (41). The disruption within the brain network could affect the function of remote regions connected with the cerebellum resulting in various symptoms other than tremor. Taken together, dysfunction in multiple nodes in the limbic system connecting the BG to the cerebellum may lead to the development of non-motor neuropsychiatric symptoms in patients with movement disorders. Future studies should use electrophysiological and neuroimaging methods to detect dysfunction in the brain network. This will elucidate the mechanisms of depression and anxiety in patients with IFD and ET. This may result in an alternative explanation to the one that states that depression and anxiety occur in patients with IFD and ET as a primary phenomenon due to disease-related disruption of neural networks. Considering that depression and anxiety are more common in patients with chronic illnesses and that the patients in our study had had IFD and ET for more than 100 months, the increased severity of depression and anxiety could be considered a secondary phenomenon due to the presence of a chronic condition (42, 43). Comparison of non-motor neuropsychiatric symptoms between *de novo* patients and healthy controls may clarify whether these symptoms have a direct relationship with the pathophysiology of IFD and ET. In our study, the severity of depression and anxiety as well as the frequency of Axis I psychiatric disorders were comparable between patients with IFD and ET, suggesting that the neuropsychiatric symptoms due to IFD and ET may share a pathophysiology attributable to dysfunctional neural networks.

We observed a significantly worse QoL in the IFD and ET groups than in the control group and a correlation between QoL and both anxiety and depression across all three groups. Interestingly, in patients with IFD, the QoL was correlated with anxiety and depression, but not with the severity of dystonia. It suggests that the QoL in patients with IFD may be more affected by neuropsychiatric symptoms than motor symptoms due to dystonia itself.

This study was subject to several limitations. First, we could not recruit the optimal number of patients that was initially calculated in the power analysis. Accordingly, the results of this study should be considered preliminary. Larger studies are required to compare the presentation of neuropsychiatric symptoms across patients with different movement disorders to elucidate their exact pathophysiology. Second, unfortunately, we did not measure tremor scale to determine the tremor severity in patients with ET. Consequently, we could not evaluate the potential correlations between motor symptoms and depression and anxiety or QoL in patients with ET. Third, although MINI is useful as a tool for the assessment of psychiatric disorders, it would not be an optimal tool designed to specifically evaluate neuropsychiatric symptoms resulting from neurological and movement disorders.

In conclusion, patients with IFD and ET has more severe depression and anxiety than healthy controls, and the increased severity is similar among patients with IFD and ET. Axis I major depressive disorder is relatively more prevalent in patients with IFD. Neuropsychiatric symptoms affect QoL regardless of the affected individual's condition, suggesting that addressing neuropsychiatric symptoms in patients with movement disorders may be crucial to improving their QoL.

## Data Availability Statement

Anonymous data related to this article will be provided by the corresponding author on reasonable request.

## Ethics Statement

The studies involving human participants were reviewed and approved by the Institutional Review Boards of Chung-Ang University Hospital (C2013231-1191) and Asan Medical Center (2014-0149). The patients/participants provided their written informed consent to participate in this study.

## Author Contributions

SL contributed to the analysis, interpretation of the data, and drafting of the manuscript. SC contributed to the study's conception, data acquisition, and critical review. H-WS contributed to the study's conception, data acquisition, data analysis and interpretation, and critical review. All authors contributed to the article and approved the submitted version.

## Conflict of Interest

The authors declare that the research was conducted in the absence of any commercial or financial relationships that could be construed as a potential conflict of interest.
